# Histopathology image classification: highlighting the gap between manual analysis and AI automation

**DOI:** 10.3389/fonc.2023.1325271

**Published:** 2024-01-17

**Authors:** Refika Sultan Doğan, Bülent Yılmaz

**Affiliations:** ^1^ Department of Bioengineering, Abdullah Gül University, Kayseri, Türkiye; ^2^ Biomedical Instrumentation and Signal Analysis Laboratory, Abdullah Gül University, Kayseri, Türkiye; ^3^ Department of Electrical and Computer Engineering, Abdullah Gul University, Kayseri, Türkiye; ^4^ Department of Electrical Engineering, Gulf University for Science and Technology, Mishref, Kuwait

**Keywords:** data science, image processing, artificial intelligence, histopathology images, colon cancer

## Abstract

The field of histopathological image analysis has evolved significantly with the advent of digital pathology, leading to the development of automated models capable of classifying tissues and structures within diverse pathological images. Artificial intelligence algorithms, such as convolutional neural networks, have shown remarkable capabilities in pathology image analysis tasks, including tumor identification, metastasis detection, and patient prognosis assessment. However, traditional manual analysis methods have generally shown low accuracy in diagnosing colorectal cancer using histopathological images. This study investigates the use of AI in image classification and image analytics using histopathological images using the histogram of oriented gradients method. The study develops an AI-based architecture for image classification using histopathological images, aiming to achieve high performance with less complexity through specific parameters and layers. In this study, we investigate the complicated state of histopathological image classification, explicitly focusing on categorizing nine distinct tissue types. Our research used open-source multi-centered image datasets that included records of 100.000 non-overlapping images from 86 patients for training and 7180 non-overlapping images from 50 patients for testing. The study compares two distinct approaches, training artificial intelligence-based algorithms and manual machine learning models, to automate tissue classification. This research comprises two primary classification tasks: binary classification, distinguishing between normal and tumor tissues, and multi-classification, encompassing nine tissue types, including adipose, background, debris, stroma, lymphocytes, mucus, smooth muscle, normal colon mucosa, and tumor. Our findings show that artificial intelligence-based systems can achieve 0.91 and 0.97 accuracy in binary and multi-class classifications. In comparison, the histogram of directed gradient features and the Random Forest classifier achieved accuracy rates of 0.75 and 0.44 in binary and multi-class classifications, respectively. Our artificial intelligence-based methods are generalizable, allowing them to be integrated into histopathology diagnostics procedures and improve diagnostic accuracy and efficiency. The CNN model outperforms existing machine learning techniques, demonstrating its potential to improve the precision and effectiveness of histopathology image analysis. This research emphasizes the importance of maintaining data consistency and applying normalization methods during the data preparation stage for analysis. It particularly highlights the potential of artificial intelligence to assess histopathological images.

## 1 Introduction

Histopathological image analysis is a fundamental method for diagnosing and screening cancer, especially in disorders affecting the digestive system. It is a type of analysis used to diagnose and treat cancer. In the case of pathologists, the physical and visual examinations of complex images often come in the form of resolutions up to 100,000 x 100,000 pixels. On the other hand, the method of pathological image analysis has long been dependent on this approach, known for its time-consuming and labor-intensive characteristics. New approaches are needed to increase the efficiency and accuracy of pathological image analysis. At this point, the digital pathology of this particular field has been completed. Digitization of high-resolution histopathology photographs allows comprehensive analysis using complex computational methods. As a result, there has been a significant increase in interest in medical image analysis for creating automatic models that can precisely categorize relevant tissues and structures in various clinical images. Early research in this area focused on predicting the malignancy of colon lesions and distinguishing between malignant and normal tissue by extracting features from microscopic images. Esgiar et al. (1) analyzed 44 healthy and 58 cancerous features obtained from microscope images. As a result of the analysis, the percentage of occurrence matrices used equals ninety percent. The first steps form the basis for more complex procedures that integrate rapid image processing techniques and the functions of visualization software. Digital pathology has recently emerged as a widespread diagnostic tool, primarily through artificial intelligence algorithms (2, 3). It has demonstrated impressive capability in processing pathology images to be advanced (4, 5). Advanced techniques, identification of tumors, detection of metastasis, and assessment of patient prognosis are utilized regularly. Through the utilization of this process, the automatic segmentation of pathological images, generation of predictions, and the utilization of relevant observations from this complex visual data were planned (6, 7).

Convolutional neural networks (convolutional CNNs) have received significant focus among various machine learning techniques in artificial intelligence, which are the techniques that are being utilized. As a result of the application of deep learning in previous biological research, it has been extensively accepted and used (8–10). The CNNs distinguish themselves from others because of their extraordinary accuracy, generalization capacity, and computational economy. Each patient’s histopathology photographs contain important quantitative data, known as hematoxylin-eosin (H&E) stained tissue slides. Attractively, Kather et al. (11) have explored the potential of CNN-based approaches to predict disease progression directly from the available H&E images. In a retrospective study, their findings underscored CNN’s remarkable ability to assess the human tumor microenvironment and prognosticate outcomes based on the analysis of histopathological images. This breakthrough showcases the transformative potential of artificial intelligence methodologies in revolutionizing the field of medical image analysis, offering new avenues for efficient and objective diagnostic and prognostic assessments.

On the other hand, in the literature, manual analysis methods are also available to classify and predict disease outcomes using the H&E images. When comparing the AI-based algorithms, traditional manual analysis generally performs lower. It is highlighted in the literature that the performance of traditional methods LBP and Haralick is poor (12, 13). These studies emphasized that deep learning is more effective in diagnosing colorectal cancer using histopathology images and that traditional machine learning methods are poor. The accuracy of LBP is 0.76 percent, and Haralick’s is 0.75. In this context, since methods such as LBP and Haralick showed low accuracy in the literature, we decided to adopt an approach other than these two methods. We chose to carry out this study with the HOG (Histogram of Oriented Gradients) method. Unlike other studies in the literature, we performed analysis using HOG features for the first time in this study. Our choice offers an alternative perspective to traditional methods and deep learning studies. The results obtained using HOG features make a new contribution to the literature. This study offers a unique perspective to the literature by highlighting the value of analysis using HOG on a specific data set.

**Table 1** provides an overview of manual analysis and AI-based studies from various literature sources. In a study by Jiang (14), a high accuracy rate of 0.89 was achieved using InceptionV3 Multi-Scale Gradients and Generative Adversarial Network for classifying colorectal cancer histopathological images. Kather et al. (6) resulted in an accuracy metric of 0.87% using texture-based approaches, Decision trees, and SVMs to analyze tissues of multiple classes in colorectal cancer histology. Other studies include Popovici et al. (15) 0.84% with VGG-f (MatConvNet library) for the prediction of molecular subtypes, 0.84% with Xu (16) CNN for the classification of stromal and epithelial regions in histopathology images, 0.83% with Mossotto (17) Optimized SVM for the classification of inflammatory bowel disease. Tsai (19) has 0.80% accuracy metrics with CNN for detecting pneumonia in chest X-rays. These results show that artificial intelligence-based classification studies generally achieve high accuracy rates. The primary emphasis of these studies revolves around artificial intelligence methods employed in analyzing histopathological images, with a particular focus on convolutional neural networks (CNNs). These networks have demonstrated exceptional levels of precision in a wide range of medical applications. These algorithms have demonstrated remarkable outcomes in cancer diagnosis and screening domains. CNNs provide substantial benefits compared to conventional approaches, owing to their ability to handle and evaluate intricate histological data. These methods excel in their capacity to detect patterns, textures, and structures in high-resolution images, thereby complementing or, in certain instances, even substituting the human review processes of pathologists. The promise of these AI-based techniques to change the field of medical picture processing is well acknowledged.

**Table 1:** Overview of the literature manual analysis and AI-based studies.

Author	Aim of Research	Method	Accuracy Metrics
Jiang (14)	Colorectal cancer histopathological images classification	InceptionV3 Multi-Scale Gradients Generative Adversarial Network	0.89
Kather (6)	Analysis of multiple classes of textures in colorectal cancer histology	Texture-based approaches Decision trees, SVMs	0.87
Popovici (15)	Prediction of molecular subtypes	VGG-f (MatConvNet library)	0.84
Xu (16)	Classifying the stromal and epithelial sections of histopathology pictures	CNN	0.84
Mossotto (17)	Classification of inflammatory bowel disease	Optimized SVM	0.83
Sena (18)	Tumor tissue classification	Custom CNN (4CL, 3FC)	0.81
Tsai (19)	Chest X-ray pneumonia detection	CNN	0.80
Shapcott (20)	Classification of nuclei	CNN based on Tensorflow “ciFar” model	0.76

## 2 Materials and methods

### 2.1 Dataset

Our research was based on the use of two separate datasets, carefully selected and prepared for use as our training and testing sets. We carefully compiled the training dataset (NCT-CRC-HE-100K) from the pathology archives of the NCT Biobank (National Center for Tumor Diseases, Germany), including records from 86 patients. The University Medical Center Mannheim (UMM), Germany (11, 21). Generated the testing dataset using (NCT-VAL-HE-7K) dataset. It included data from 50 patients. We obtained the datasets from open-source photographs after carefully removing them from formalin-fixed paraffin-embedded tissues of colorectal cancer. The dataset we used for training and testing consisted of 100,000 high-resolution H&E (hematoxylin and eosin) images.

From these images, we selected 7180 non-overlapping sub-images, also known as sub-images. Each of these sub-images measures 0.5 microns in thickness and boasts dimensions of 224x224 pixels. The richness of our dataset is further highlighted by the inclusion of nine distinct tissue textures, each encapsulating the subtle difficulties of various tissue types. These encompass a broad spectrum, from adipose tissue to lymphocytes, mucus, and cancer epithelial cells. **Table 2** meticulously presents the distribution of images within the test and training datasets, segmented by their respective tissue classes. For instance, we meticulously assembled a training dataset featuring a robust 14.317 samples within the colorectal cancer tissue class. Simultaneously, the testing dataset for this class comprises 1.233 samples. These detailed statistics play a crucial role in providing readers with a comprehensive understanding of the data distribution and the relative sizes of each class within the study, forming the foundation for our subsequent analyses and model development.

**Table 2:** The number of H&E images in the test and training set used in the study.

Classes	Number of Training	Number of Tests
**ADI**	10.407	1.338
**BACK**	10.566	847
**DEB**	11.512	339
**LYM**	11.557	634
**MUC**	8.896	1.035
**MUS**	13.536	592
**NORM**	8.763	741
**STR**	10.446	421
**TUM**	14.317	1.233

**Figure 1:**
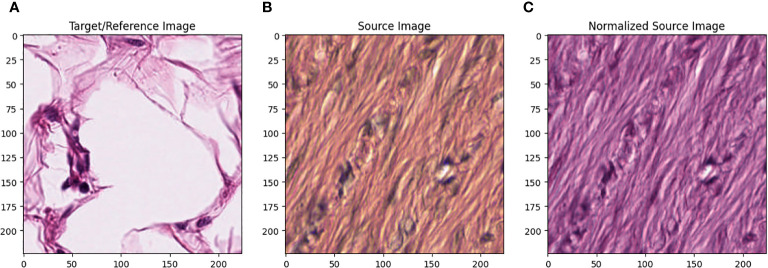
**(A)** Target/reference image, **(B)** source image, and **(C)** normalized source image.

All images in the training set were normalized using the Macenko method (22). **Figure 1** describes the effect of Macenko normalization on sample images. The torchstain library (23), which supports a PyTorch-based approach, is available for color normalization of the image using the Macenko method.

**Figure 2:**
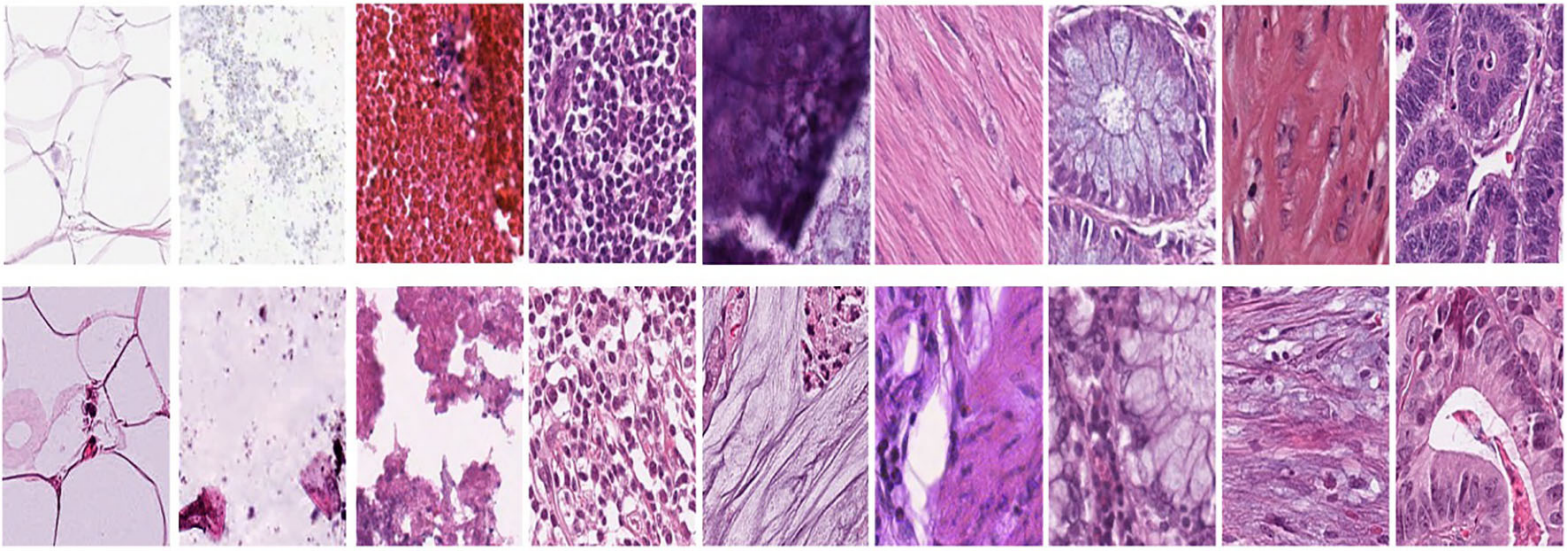
First row represents Adipose (ADI), background (BACK), debris (DEB), lymphocytes (LYM), mucus (MUC), smooth muscle (MUS), normal colonic mucosa (NORM), cancer-associated stroma (STR), colorectal adenocarcinoma epithelium (TUM) and the second row data set was obtained by applying normalization to the same tissue examples.

**Figure 1A** represents this method’s target/reference image, while **Figure 1B** represents the source images. Macenko normalization aims to make the color distribution of the source images compatible with the target image. In the example shown in the figure, the result of the normalization process applied on the source images (**Figure 1B**), taking the target image (**Figure 1A**) as a reference, allows us to obtain a more consistent and similar color profile by reducing color mismatches, as seen in **Figure 1C**. This will make obtaining more reliable results in machine learning or image analytics applications possible. Normalization was performed on the dataset on which the model was trained, and applying this normalization to the test set can increase the model’s generalization ability. However, the test set represents real-world setups and consists of images routinely obtained in the pathology department. Therefore, since these images wanted to train a clinically meaningful model with different color conditions, they were not applied to the normalization test set. In this way, we also investigated the effect of applying color normalization on classifying different types of tissues. The original data set shown in **Figure 2** first row from nine different tissue samples has substantially different color stains; however, **Figure 2** second row shows their normalized versions. These images are transformed to the same average intensity level.

### 2.2 Manual analysis algorithm

In traditional manual analysis, the classification process was emphasized by extracting HOG (Histogram of Oriented Gradient, Histogram of Directional Gradients) features. HOG features represent a class of local descriptors that extract crucial characteristics from images and videos. They have found typical applications across various domains, encompassing tasks such as image classification, object detection, and object tracking (24, 25).

The following parameters were used to extract HOG features: The number of orientations is 9. This is the number of gradient directions calculated in each cell. The cells per pixel are: Each cell consists of 10x10 pixels. The blocks per cell: Each block contains 2x2 cells. Rooting and Block Normalization: Using the `transform_sqrt=True` and `block_norm=“L1”` options, rooting and L1 norm-based block normalization were performed to reduce lighting and shading effects. The resulting features are more robust and amenable to comparison, especially under variable lighting conditions. This can improve the model’s overall performance in image recognition and classification tasks. Using these parameters increases the efficiency and accuracy of the HOG feature extraction process, thus ensuring high performance in colorectal cancer tissue classification.

**Figure 3:**
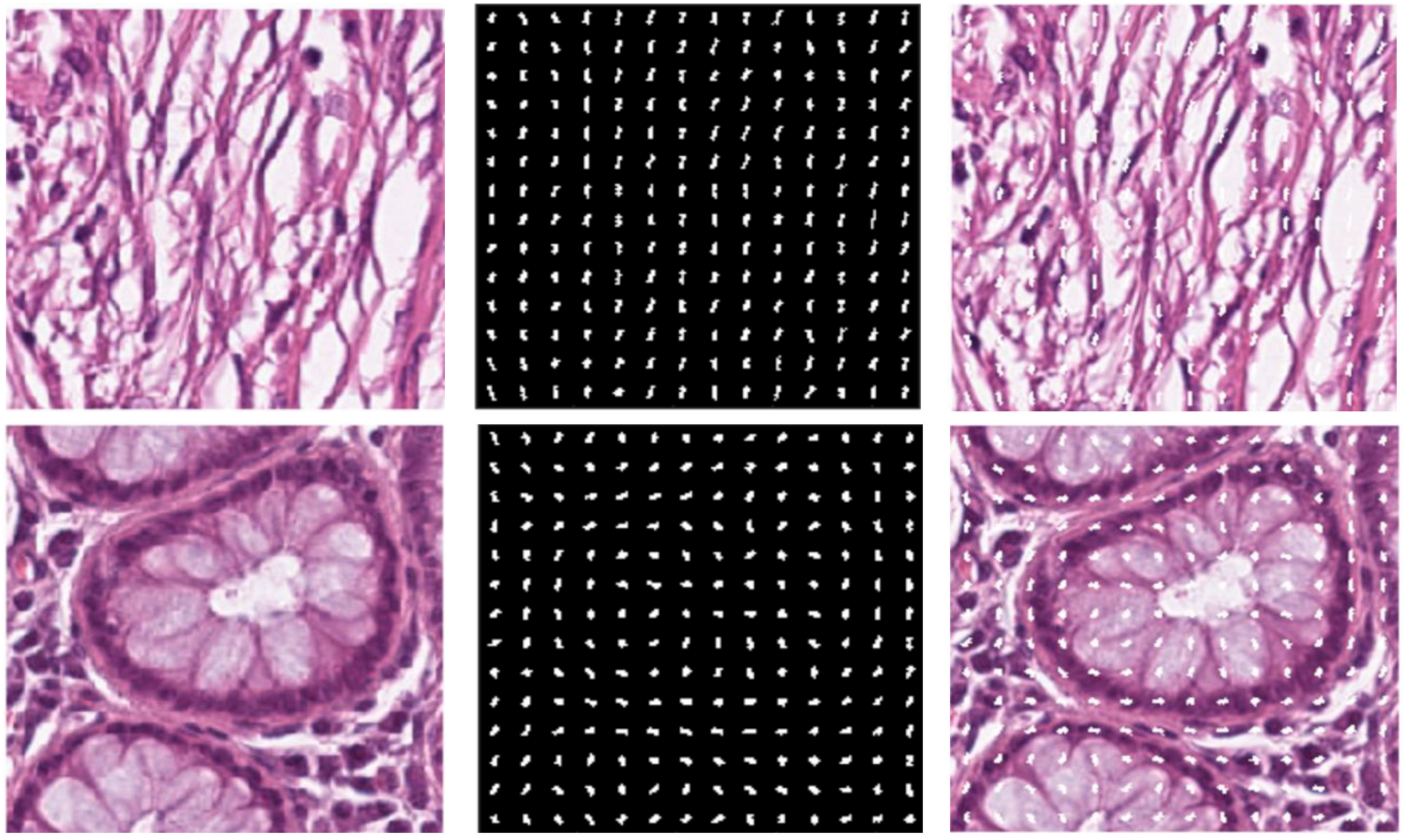
**(A)** Original image **(B)** Extracted HOG features from the image **(C)** HOG features shown on the original image.

We chose HOG features, one of the local descriptors, because HOG processes the image by dividing it into small regions called cells. As illustrated in **Figure 3**, the cells are created with the original images. In the created cells, the gradients of the pixels in the x-direction (Sx) and the y-direction (Sy) are calculated using Equation 1. The gradient direction θ is computed using the computed gradients in Equation 2 (26).


(1) $$S=\;\sqrt{S_{x}^{\;2}+S_{y}^{\;2}}$$

(2) $$\text{θ}=\;\tan^{-1}\left(\frac{S_{x}}{S_{y}}\right)$$

After calculating the gradients, the histograms are calculated, and these histograms are combined to form blocks. Normalization is performed on the blocks to avoid lighting and shading effects. The study involved a comprehensive analysis of the image set, where all images were initially standardized to a dimension of 224x224. This standardization was conducted to enhance the classification performance of the features derived from these images. To achieve this, a bilinear interpolation method was applied, resizing the images to a standardized dimension 200x200. The decision to reduce the image dimensions from 224x224 pixels to 200x200 pixels was taken to optimize the calculation time. In particular, a 224x224 image produces 7200 feature vectors, while a 200x200 image performing the same process produces 5832 feature vectors. The feature vector formula is explained as follows (Equation 3).


(3) $$Feature\;vector\;size=N\;\times \left(\frac{W}{C_{W}}-CpB_{X}+1\right)\times \left(\frac{H}{C_{H}}-CpB_{y}+1\right)\times Orientations\times CpB_{x}\times CpB_{y}\;$$

In Equation 3, N is the number of pixels in the image, W and H are the image’s width and height, respectively, $C_{W}$ cell weight and $C_{H}$ cell height defines cell dimensions, $CpB_{x}$ and $CpB_{y}$ are the number of cells per block, representing the number of directions calculated for each cell in the HOG feature vector. Not only does dimensionality reduction have the advantage of reducing computational time, but smaller-sized feature vectors can potentially reduce memory usage and the overall complexity of the model. This is due to optimizing model training and prediction times, especially when working on large data sets.

This study preferred the Random Forest (RF) algorithm as the machine learning model for colorectal cancer tissue classification. RF is one of the ensembles learning methods and creates a robust and generalizable model by combining multiple decision trees. The main reason for this choice is that RF performs highly on different data sets. It can work effectively on complex and multidimensional data sets. RF can operate effectively on large data sets and high-dimensional feature spaces. RF is resilient to noise and anomalies in the data set. It can also evaluate relationships between variables, increasing the stability of the model. RF can deal with overfitting problems, preventing the model from overfitting the training data. These features support the suitability of the RF algorithm for colorectal cancer tissue classification. As a result of preliminary tests and analyses, it was decided that RF was the most suitable model. This choice is intended to obtain reliable results.

RF applications are practical biomedical imaging and tissue analysis tools with features such as high-dimensional data processing ability, accuracy, and robustness. Challenges to this implementation, such as computational efficiency, potential overfitting, and especially interpretability and explainability, are also significant. It was stated that the algorithm was possible and was considered to increase security, especially for the future of medicine and clinical research. It is interesting to note that comprehensive feature selection plays a critical role in learning and comprehensively makes the consolidated results more accurate and robust. This is extremely important in increasing efficiency in medicine and clinical research (27, 28).

### 2.3 AI-based automation

In this study, a remarkable CNN architecture was developed for the image classification problem. The developed model aimed to achieve high performance with less complexity through specific parameters and layers. We used a simple CNN-based architecture and trained it using the same H&E images, the same data we used in the manual analysis part. We aim to compare the performance of manual analysis and AI-based automation methods in classifying colorectal cancer tissue images. **Table 3** shows the parameter and structure information of the CNN model.

**Table 3:** Details, complexity, and hyperparameters of the simple CNN architecture we build for the AI automation approach.

CNN layers	Parameters and explanations	Complexity
1 Image Input	28x28x3 images with ‘zscore’ normalization	Low - preprocessing step
2 Convolution	8 3x3 convolutions with stride [1 1] and padding ‘same’	Moderate – only eight filter
3 Batch Normalization	Batch Normalization	Low to moderate
4 ReLU	ReLU	Low
5 Max Pooling	2x2 max pooling with stride [2 2] and padding [0 0 0 0]	Low
6 Convolution	16 3x3 convolutions with stride [1 1] and padding ‘same’	Moderate
7 Batch Normalization	Batch normalization	Low to moderate
8 ReLU	ReLU	Low
9 Max Pooling	2x2 max pooling with stride [2 2] and padding [0 0 0 0]	Low
10 Convolution	32 3x3 convolutions with stride [1 1] and padding ‘same’	Higher due to the increased number of filters
11 Batch Normalization	Batch normalization	Low to moderate
12 ReLU	ReLU	Low
13 Fully Connected	Three fully connected layer	High
14 Softmax	softmax	Low
15 Loss function	crossentropyex	Low

In the manual analysis section, steps were taken to extract features from images and train the model using these features. Nevertheless, we used images directly as input in the AI automation part, then created a model suitable for the purpose and carried out the training process. In the AI automation approach, we used local filters, intermediate steps, and a multilayer artificial neural network model to train the base CNN model (**Table 3**) (10). This table explains the layers, structures, and hyperparameters of the CNN model used in the AI automation section. This model includes direct use of images and essential operations such as sequential convolution, batch normalization, and ReLU activation. Finally, it uses the classification of results with softmax activation and a cross-entropy-based loss function. This model reflects a complex structure aimed at classifying colorectal cancer tissue images. This approach has played an important role in comparing the performance of manual analysis and AI automation methods.

This AI automation model is designed to extract and classify features in histopathological images. In the first layer, the model gets input from color (RGB) images of 28x28 pixels. Input images are processed with ‘zscore’ normalization, which brings the mean of the data to zero and its standard deviation to one. The model’s architecture then includes a series of convolutional layers, batch normalization, and ReLU activation functions. Convolution layers move over the image to extract feature maps and highlight important features. Batch normalization helps train the network faster and provides more stable performance. ReLU activation functions filter out negative pixel values, increasing the learning ability of the model. Maximum pooling layers shrink the feature maps and increase the model’s scalability. As a result of these layers, the model includes high-level features such as learning and increasing complexity. Finally, the model uses fully connected layers to assign learned features to specific classes and uses the softmax activation function to make the results more consistent.

On the other hand, its existence allows a probability distribution to be provided for each class. The cross-entropy loss function optimizes the learning of the model with accurate classification labels and manual analysis techniques, which are examples of techniques that can be used to optimize the process. As a result of this model, AI automation can improve feature extraction and classification capabilities in histopathology image classification tasks.

The study selected parameters to train the model based on starting values commonly accepted in the literature (29). In the early stages of the training process, researchers attempted to achieve gradual improvement by choosing a varying initial learning rate. The researchers determined that the maximum number was fifty and presented the maximum number as one hundred, allowing the model to follow the training data over a long period. At each epoch, the model rearranged the training data to produce a more comprehensive and independent representation unaffected by prior learning. We chose a batch size ranging from 32 to 64 to ensure uniform processing of the samples. We used these parameters to select validation data and determine the evaluation frequency. Continuous evaluation of the model throughout the training process is not only guaranteed but also guaranteed. By eliminating cases of overfitting, the model achieved greater generalizability, and more reliable results were confirmed. We conducted rigorous testing and used a trial-and-error approach to determine these parameters to monitor the model’s performance. Research findings show that the selected parameters yield the best results, and the model effectively facilitates learning from the dataset.

During the last training session, we carefully determined the exact parameter values that led to the successful training of the model: We set the initial learning rate to 0.01 and the maximum number of epochs to 50. Blending is the process of combining data. We carried out from different sources. Every complete pass across the entire dataset is performed at every epoch. This is intended to ensure the size is set to 64 during the process. You will also see that 64 data samples are processed together. He managed to complete the task for twenty days successfully. The model in question is a specific learning rate that uses the number of epochs and other parameters that reflect a unique scenario to be trained. We carefully chose these parameters for the model to achieve the necessary level of success and assure the best possible fit to the data set.

In this study, the researchers developed a convolutional neural network (CNN) model to improve their ability to extract essential features and classify histopathology images. The model takes 28x28x3 RGB images as input, processing them with ‘zscore’ normalization. The model structure includes three 3x3 convolution layers containing 8, 16, and 32 filters. The ReLU activation function was used after each convolution layer. Additionally, there are 2x2 sized Max Pooling layers following each convolution layer. In the final stages of the model, there are three fully connected layers; the softmax activation function was preferred as the last layer. In terms of training parameters, explained in **Table 4**, the model is initially trained with a learning rate of 0.01 and works on the data set for a maximum of 50 epochs. Data shuffling is applied at the end of each epoch, and 64 is selected as the batch size. The performance and generalization ability of the model are continuously monitored with validation dataset evaluations performed every 20 epochs. Determining these parameters ensures that the model adapts to the data set most appropriately and reaches the desired level of success, and also helps the model avoid possible problems such as overfitting during the training process. With this configuration, the model has the necessary feature extraction and classification capabilities to produce effective and accurate results in histopathological image classification.

**Table 4:** The CNN-based automation model training parameters.

Training Parameter	Value
Initial Learning Rate	0.01
Maximum Number of Epochs	50
Data Shuffle	After each epoch
Batch Size	64
Verification Data and Evaluation Frequency	Every 20 epochs.

The cross-entropy loss (Equation 4) calculates the difference between the probability distribution that the model predicts and the probability distribution of the actual labels. The formula for binary classification is as follows:


(4) L(y,p)=−(ylog(p)+(1−y)log(1−p)) 

In Equation 4, L represents the loss function, y represents the actual label value (1 or 0), and p represents the probability predicted by the model. The formula for multiclass classification is usually:


(5) $$L=-\sum_{c=1}^{M}\left(y_{o,c}\text{log}(p_{o,c}\right)\;$$

In Equation 5, M denotes the number of classes, $y_{o,c}\;$ is the binary representation (1 or 0) indicating o whether or not the instance belongs to class c. If that instance belongs to class c, this value is 1; otherwise, it is 0. $p_{o,c}$ is the probability that the model predicts that sample o belongs to class c.

One of the reasons for choosing cross-entropy loss is direct probability evaluation. Cross-entropy directly evaluates how close the probabilities produced by the model are to the actual labels, making it a natural choice for classification problems. Faster convergence is another reason for choosing it. This function helps the model converge faster and more efficiently during gradient-based optimization, mainly thanks to the logarithmic component. Cross entropy works on probabilities directly affecting the model’s performance in classification problems. This directly improves the model’s ability to predict class labels accurately. Cross-entropy loss imposes a significant penalty on incorrect predictions, especially in cases where the model is very confident in its incorrect prediction. This prevents the model from making incorrect predictions that are overconfident.

The CNN model developed in this study was evaluated in terms of computational complexity depending on the model’s architecture and learning process. Our model is analyzed for time and space complexity, considering factors such as interlayer transitions and filter sizes. Each convolution layer has $O\left(k.n^{2}\right)$ time complexity to extract features between adjacent pixels. Here k represents the filter size and $n^{2}$ represents the size of the image. Our model has a time complexity of $O\left(k.n^{2}.d\right)$ for one training epoch, where d is expressed as depth (number of layers). Space complexity is directly related to weight matrices and feature maps and specifies the amount of memory the model requires. As a result of this study, it was observed that the model can scale effectively in large data sets and exhibit high performance in practical applications.


(6) #parameters=(FilterHeight * FilterWidth * InputChannels+1) * NumberofFilters   

The number of parameters for the first convolutional layer can be calculated using Equation 6, (3 * 3 * 3 + 1) * 8, and obtain 224. This operation is computationally exhaustive but with only eight filters, therefore, complexity is moderate.

### 2.4 Model evaluation

This study used two essential methods to evaluate model performance and obtain reliable results. First, the metrics used to evaluate the model’s performance and the reasons for choosing these metrics are stated. Then, it details why the 10-fold stratified cross-validation method was preferred during the training of the manual analysis model.

It is crucial to choose the right metrics to evaluate model performance. In this study, commonly used metrics such as accuracy, precision, recall, and F1 score were preferred to measure the model’s classification performance and explained in Equations 7–10, respectively. Accuracy is the ratio of correctly classified samples to the total number of samples. That is, it refers to the ratio of true positives and true negatives to the total samples. Precision shows the proportion of samples predicted to be positive that are positive. It refers to the ratio of true positives to total positive predictions. Recall shows the ratio of true positives to the total number of positive samples. While accuracy refers to overall correct predictions, precision and sensitivity evaluate the model’s performance in more detail, especially in unbalanced class distributions. F1 score is a performance measure calculated as the harmonic mean of precision and recall values. In unbalanced class distributions, the F1 score is used to evaluate the model’s performance on both classes in a balanced way. In unbalanced class distributions, especially in cases where the majority class has more samples, the model must make true positive and true negative predictions in a balanced way.


(7) $$Accuracy=\;\frac{TP+\;TN}{TP+\;TN+FP+FN\;}\;\;\;$$

(8) $$Precision\;\left(P\right)=\;\frac{TP}{TP+FP}\;\;\;\;\;\;\;\;\;\;\;$$

(9) $$Recall\;\left(R\right)=\;\frac{TP}{TP+FN}\;\;\;\;\;\;\;\;\;\;\;\;\;\;$$

(10) $$F1\;score=2\times \frac{P\times R}{P+R}\;\;\;\;\;\;\;\;\;\;\;\;\;\;\;$$

For the model to generalize reliably and to avoid overfitting problems, the 10-fold stratified cross-validation method was preferred. This method divides the dataset into ten equal folds and uses each fold as validation data while training the model using the remaining 9-fold as training data. This process continues until each fold is used as validation data. Stratification preserves the class proportions in each tissue type, allowing the model to learn and evaluate equally in each class. This ensures the model can generalize over various data samples, allowing us to obtain reliable results.

In this study, a paired t-test was used to determine that the classification results of the proposed approach were not obtained by chance. The significance of the difference in the overall accuracy achieved by the models was assessed by calculating Paired t-test p values for the overall accuracy of the classification performance achieved by the models. Statistical analysis was performed using the stats module of the Scipy library (version 1.11.3) (30) of Python (version 3.8). P values less than 0.05 were considered statistically significant. The p-value of the paired t-test is explained in the results part.

Within the scope of this research, a convolutional neural network (CNN) model was developed using MATLAB R2023a and AI automation Toolbox 16.3 versions for AI automation tasks. MATLAB was used for data manipulation, training, and evaluation of results. The manual analysis classification model was built using Python 3.8 along with the deep learning model. This model is integrated with scikit-learn (v0.24.2) and NumPy (v1.20.3) libraries. Python has been used in feature extraction and classification tasks. In terms of hardware, the study was run on a personal computer, MacBook Air, with an Apple M1 chip with eight cores and 8 GB of memory. macOS Big Sur (v11.2.3) was used as the operating system. These hardware and software configurations increase the understandability of the methodology, ensure the reproducibility of results, and facilitate comparability of similar studies.

Our research used a large dataset of H&E images of various classes. Specifically, the dataset contains 10.407 ADI, 10.566 BACK, 11.512 DEB, 11.557 LYM, 8.896 MUC, 13.536 MUS, 8.763 NORM, 10.446 STR, and 14.317 TUM images for training, and the test sets are detailed in **Table 2**. Remarkably, training our CNN model on a dataset of approximately 100.000 images was completed in 200 seconds, demonstrating that our approach is practical even with large-scale data.

## 3 Results

Our classification study started with a comprehensive examination aimed at distinguishing normal and tumor tissues selected from a diverse collection of nine distinct tissue types. This initial phase of our research involved utilizing Histogram of Oriented Gradients (HOG) features extracted from the images. We employed the Random Forest classifier model to assess the effectiveness of this approach.

For a more visual representation of our results, figures explain the confusion matrices derived from the CNN model and the manual analysis results in the supplementary part. This visual insight provides a comprehensive view of the classification performance.

These complex confusion matrices in high-dimensional datasets provide in-depth information about the model’s ability to classify tissues. In particular, the ADI class has a high accuracy rate in both normalized and non-normalized scenarios. The LYM class exhibits low sensitivity when the data is not standardized. Furthermore, the TUM class demonstrates high accuracy and sensitivity, especially in normalized situation. It is clear from this that the model can accurately identify cancerous tumors.

On the other hand, the STR class has low accuracy and sensitivity, particularly in a non-normalized situation. This may indicate that this texture is more challenging to categorize than other textures. As a consequence of this, these matrices are an essential instrument for assessing the performance of the model on various types of tissue and for gaining an understanding of the model’s strengths and shortcomings. In addition, it sheds light on the challenges associated with the classification of uncommon classes and the potential for normalization to ease these challenges. These findings potentially provide valuable direction for future work to enhance the model and improve its performance.

**Figure 4:**
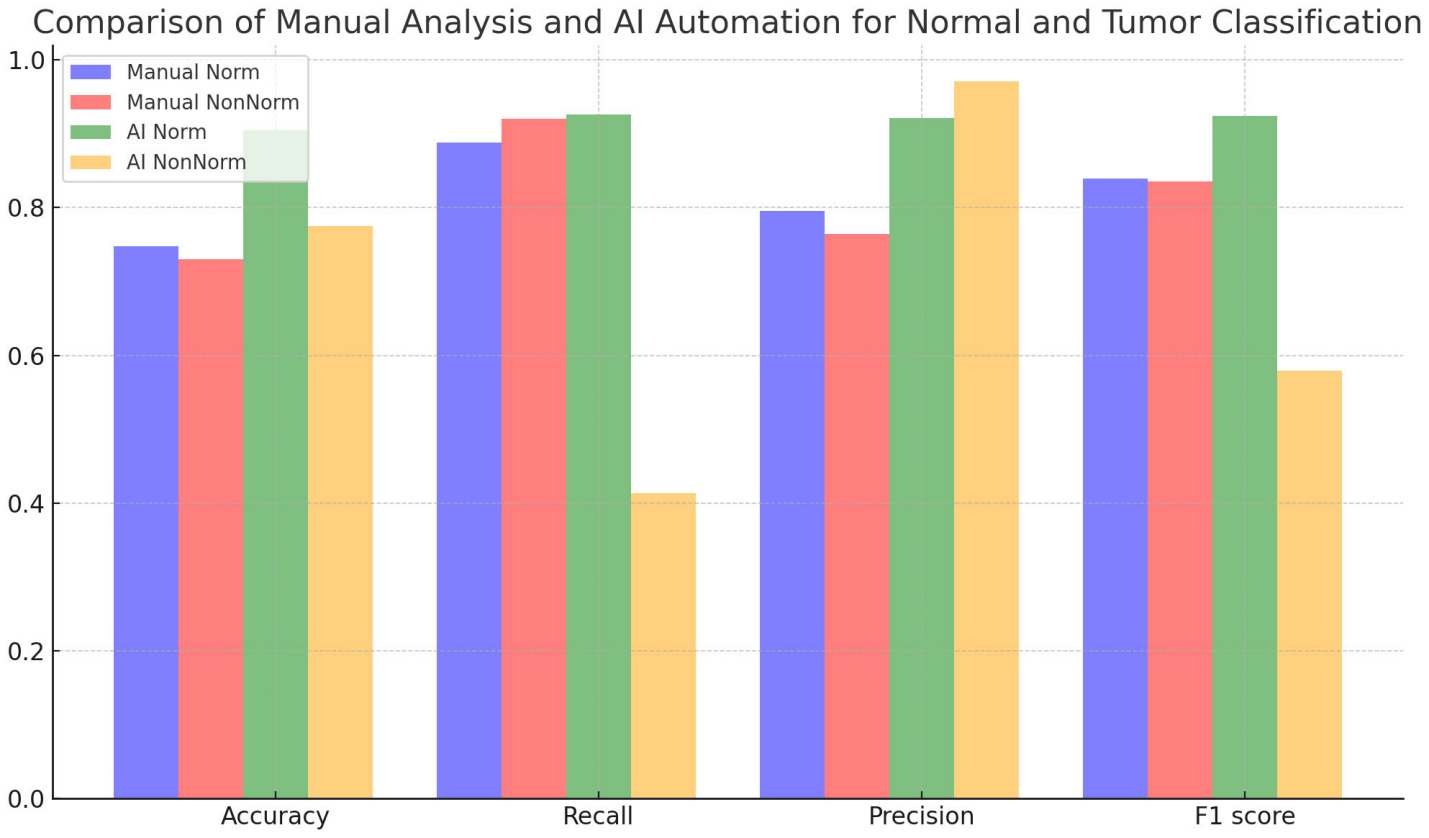
Comparison of manual analysis and AI automation for normal and tumor classification.

**Table 5** and **Figure 4** evaluate the effect of normalization in four different cases, with and without normalization, and includes accuracy results obtained with manual analysis and AI automation models for different tissues. When normalized, manual analysis accuracy for N-T tissues increased from 0.75 to 0.91, which was highly significant (p=7,72x10^-41^). Without normalization, accuracy increased from 0.74 to 0.78 for the same comparison, which was statistically significant (p=0.0033). Additionally, the effect of normalization varies between tissues; Significant changes in accuracy are observed depending on the tissues (p<0.05). According to the analysis results, the p-value is under 0.05, a statistically significant level. This shows that the differences between the analyzed results are unlikely to be coincidental, and the reliability of the findings is statistically supported.

**Table 5:** Accuracy results (N, Normal; T, Tumor).

Normalization	Tissue type	Manual analysis	AI automation	Significance
**No**	N-T	0.74	0.78	Yes
	Nine tissues	0.41	0.86	Yes
**Yes**	N-T	0.75	0.91	Yes
	Nine tissues	0.44	0.97	Yes

**Table 6** compares the classification results performed by manual analysis with the effect of normalization. Although normalization increased accuracy, this was not statistically significant (p = 0.2108). While recall decreased slightly with normalization, this decrease is statistically significant (p = 0.0005). Precision increases with normalization, which is statistically significant (p=0.0162). Regarding the F1 score, the effect of normalization is not statistically significant (p = 0.7144).

**Table 6:** Normal and tumor classification evaluation metrics with and without normalization using manual analysis.

	Norm	NonNorm	Significance
Accuracy	0.7475	0.730	No
Recall	0.8879	0.9204	Yes
Precision	0.7958	0.7641	Yes
F1 score	0.8393	0.8350	No

**Table 7** contains an analysis in which normal and tumor classifications performed by AI automation are evaluated under the influence of normalization. Normalization caused statistically significant improvements in accuracy, recall, precision, and F1 score metrics (p=1.08x10^-28^, p=1.66x10^-26^, p=3.87x10^-12^, p=2.01x10^-14^), respectively. These results show that normalization is efficacious in improving AI automation-based classification performance.

**Table 7:** Normal and tumor classification evaluation metrics with and without normalization using AI automation.

	Norm	NonNorm	Significance
Accuracy	0.9048	0.7751	Yes
Recall	0.9262	0.4130	Yes
Precision	0.9217	0.9714	Yes
F1-score	0.9239	0.5795	Yes

**Figure 5:**
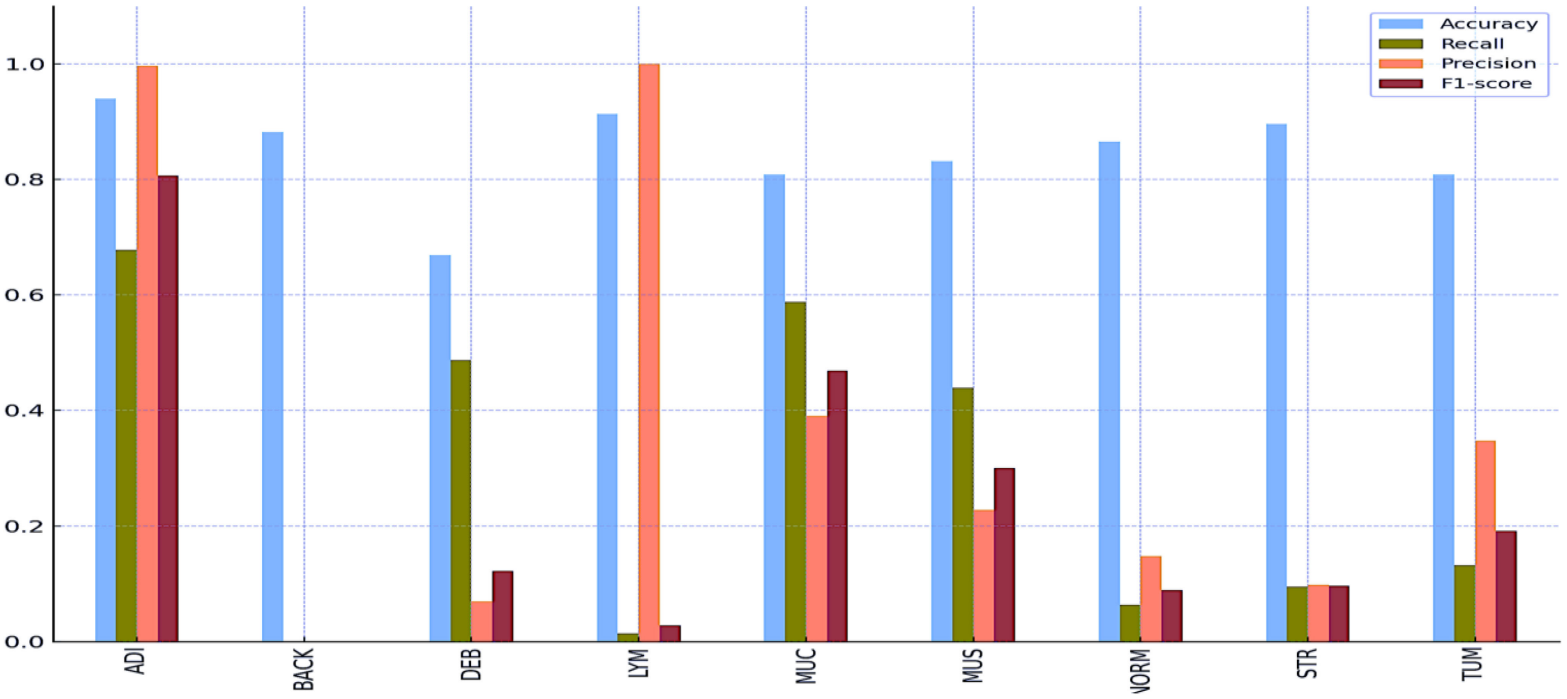
Multi-class classification results without normalization using AI automation.

**Figure 6:**
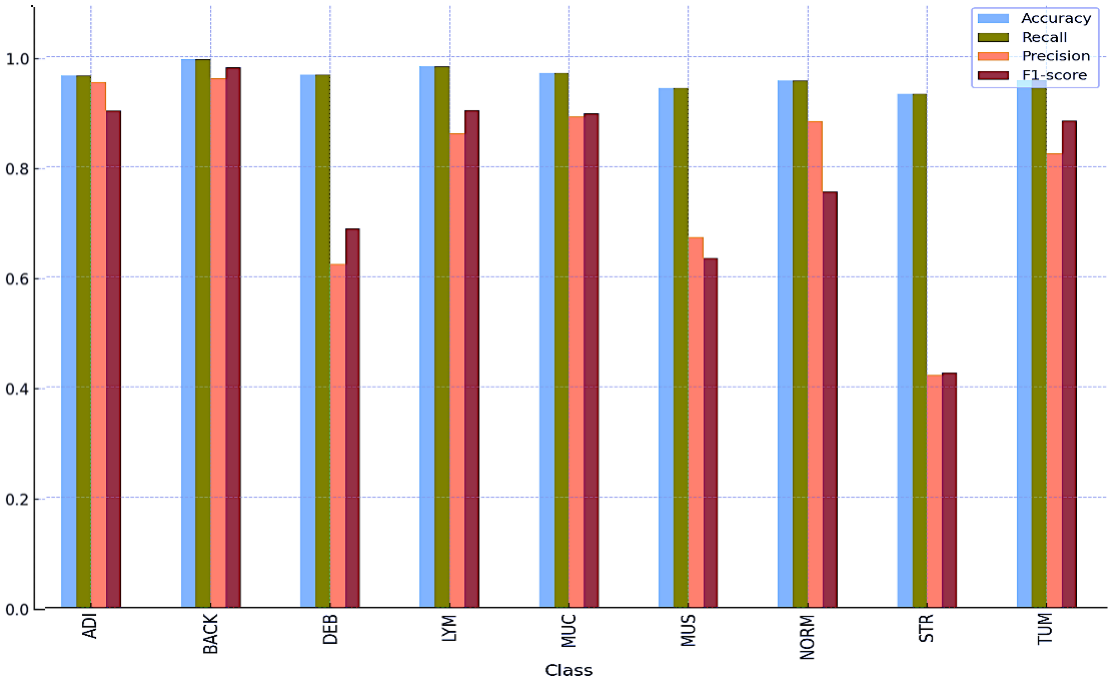
Multi-class classification results after normalization using AI automation.

**Tables 8** and **9** and **Figures 5** and **6** also include the multiple classification results using AI automation for all classes before and after normalization was applied. With the application of normalization, the increases observed in the evaluation metrics of especially low-performing classes show that the model significantly improves its classification. **Table 8** contains two values of particular significance: 0 and NaN (Not a Number) within the “BACK” category. This can indicate that the class failed or did not manage to calculate for specific metrics. As shown in **Table 9**, an overall performance improvement was observed after updating these NaN and 0 values. As a result of normalization, the performance of each class became more consistent and equal. This suggests that the model exhibits enhanced robustness and consistency in its output to normalization.

**Table 8:** Multi-class classification results without normalization using AI automation.

	Accuracy	Recall	Precision	F1-score
ADI	0.9396	0.6779	0.9967	0.8069
BACK	0.8820	0	NaN	0
DEB	0.6689	0.4867	0.0697	0.1219
LYM	0.9130	0.0142	1.000	0.0280
MUC	0.8085	0.5874	0.3907	0.4693
MUS	0.8311	0.4392	0.2279	0.3001
NORM	0.8656	0.0634	0.1478	0.0888
STR	0.8955	0.0950	0.0978	0.0964
TUM	0.8084	0.1322	0.3475	0.1915

**Table 9:** Multi-class classification results after normalization using AI automation.

	Accuracy	Recall	Precision	F1-score
ADI	0.9652	0.9652	0.9533	0.9015
BACK	0.9951	0.9951	0.9603	0.9798
DEB	0.9671	0.9671	0.6235	0.6878
LYM	0.9819	0.9819	0.8600	0.9025
MUC	0.9699	0.9699	0.8911	0.8963
MUS	0.9429	0.9429	0.6723	0.6339
NORM	0.9557	0.9557	0.8825	0.7543
STR	0.9320	0.9320	0.4219	0.4259
TUM	0.9567	0.9567	0.8237	0.8830

## 4 Discussion

Intending to classify histopathology images accurately, this study attempts to analyze the operational mechanisms of various methods. Distinguishing between normal and tumor tissues and categorizing tissues into nine distinct groups were this study’s two primary classification objectives. The analysis results also assessed the effects of different normalization methods. We conducted a comparative analysis between conventional manual analysis and deep learning methodologies powered by AI. Our findings were considerably enhanced through the implementation of normalization methods; in particular, the “Normal-Tumor” and “Nine Categories” classification tasks yielded substantially higher accuracy rates after normalization. On the other hand, it demonstrates the critical importance of improving the overall effectiveness and maintaining the data integrity of our models.

AI methods offer superior performance compared to manual analysis and AI-powered automation. It demonstrates the ability to distinguish between normal and tumorous tissues in histopathological images, as evidenced by the AI automation’s 91% accuracy rate in its classification of “normal-tumor”. AI methods showed superior performance on the “Nine Categories” task, where texture posed a more significant challenge than human analysis. In this particular instance, the accuracy rates dropped due to the complexity not being present. On the other hand, the integration of artificial intelligence methodologies has increased accuracy rates despite the increasing complexity of this particular situation, which is a contradiction. The results of an automated histopathology image analysis reveal the tremendous potential of artificial intelligence-based methods utilized within the otolaryngology field. It is vital throughout the data processing phase. This is the objective of this study.

In terms of application, the cross-entropy loss function accelerated the model’s training process and gave positive results. As a result of this function, the model’s stabilization and classification accuracy increased despite the data sets’ asymmetric structure. Cross-entropy loss increased the generalization capacity of the model by reducing entropy loss, further reducing the potential for overfitting. The evaluation of both included test datasets also showed that the model had improved predictive capability and accuracy when applied to unobserved data. It was determined that cross-entropy was the most appropriate loss function for this example through experiments with alternative functions. For example, cross-entropy provided faster convergence and higher classification accuracy compared to mean square error loss, faster convergence, and higher classification accuracy compared to error loss.

At the same time, the model’s efficiency was increased by making additional adjustments to the parameters of the loss function. As a result of the utilization of the model, the optimization of the hyperparameters of the loss function is performed on the result. This allows for optimizing the loss function, the optimization of the hyperparameters, and the optimization of the recall ratio. In the context of the model, it performed exceptionally well, even when faced with complex data types. Given the significant improvement in overall performance that this particular option can bring, it is arguably the best option for our model other than the entropy loss.

It reveals a significant difference in cases where normalization is applied, which is the subject of the verification of statistical analysis in these tables. Specifically, the various texture classification results in **Table 5** show that normalization has a significant effect. An analysis and an AI automation modeling review show that normalization significantly affects both scenarios, which is the goal of this investigation. A comparison was made between the classification results of samples with and without normalization, which can be found in **Tables 6**, **7**. It shows that normalization significantly improves the classification performance, which is statistically significant. The results presented in **Table 9** are particularly relevant to the AI automation model and show that normalization has a more pronounced effect. In summary, statistical analyses prove that the improvements in the classification performance of the proposed method are not coincidental and that normalization is an important determinant.

As a result of this artificial intelligence-based model, it is considered a good alternative as it performs as well as and better than the more complex models described in the literature. The dataset’s features, the number of classes, and the complexity of the proposed model are all used in this context. In summary, this research contributes significantly to the ongoing discourse surrounding comparing established approaches to the automatic categorization of histopathology images (31, 32). The results highlight the indisputable advantages of AI automation and the critical importance of data normalization and interpretation; these should be considered in further research in this area. Results from this research contribute significantly to advancing knowledge in this field and can potentially guide subsequent advances in automated histopathology image analysis. In the relevant scientific field, the findings can serve as a handbook for researchers facing similar conditions.

An example would be the LBP and Haralick methods, which may have limitations when processing high-resolution histopathological images. These images may be better suited to artificial intelligence algorithms that can process large data sets and identify complex patterns, and artificial intelligence. Unlike traditional methods, artificial intelligence algorithms with advanced feature extraction and learning capabilities can extract features more comprehensively. It can perform more comprehensive analysis by gaining information about complex relationships and patterns within the data set. CNN and other artificial intelligence algorithms are examples of websites that provide such algorithms. Using variable data types can pose challenges and face difficulties adapting to traditional approaches often designed for specific situations.

In contrast, AI algorithms exhibit greater adaptability and can expertly navigate diverse data types and dynamic environments. AI algorithms can be superior to traditional methods in terms of automation and speed. This provides a significant advantage, especially in time-consuming and labor-intensive procedures, for example, large-scale histopathological image analysis.

There are some limitations to this study design. First, the data set’s size and diversity can affect the model’s generalization ability. Access to a more extensive and diverse data set can help the model achieve more robust and overall performance. Additionally, the imbalance between classes can cause difficulties in classifying infrequent classes. This imbalance can affect the learning curve of the model. It may be recommended to overcome these limitations using more balanced data sets in future studies. This study reveals several future work and application opportunities that can be considered in a broader context. First, how the model will perform on other medical imaging datasets can be further evaluated. Studies focusing on a similar classification task in different medical fields could evaluate the general applicability of the model.

Additionally, application studies can focus on how the proposed model can be integrated into more specific clinical applications. This can evaluate the usability and effectiveness of the model on real patients in clinical applications. In future studies, integrating the model into real-time applications and how it can contribute to patient care can be investigated in more detail. To validate the model’s performance on other medical imaging datasets, evaluate its broad applicability in many medical domains, and investigate integration into clinical applications, the paper makes recommendations for further work. It implies that more research might look into the model’s potential for real-time use and how it can improve patient care.

AI-based automation has emerged as a significant development in healthcare technologies in recent years. Developing and implementing AI systems contribute to transforming clinical practices, especially in digital pathology. However, the effective integration of these technologies and their acceptance in clinical settings is closely related to the traceability, explainability, and interpretability of the results provided by AI systems (33, 34). Current trends highlight the compliance of AI applications with standards of transparency and accountability, especially within the framework of the European Union’s Medical AI laws. In this context, the ability of AI systems to explain the reasons and mechanisms underlying the decisions they make is critical for clinicians’ acceptance of these technologies. Evans et al. (35) emphasize that the usability of AI-based systems in clinical settings is directly related to the ability of these systems to explain and interpret their results. Therefore, when evaluating the potential of AI-based automation in our work, we found it essential to focus on the interpretability and explainability of these technologies. To better understand the role of AI systems in clinical decision-making processes and integrate these technologies into clinical environments, algorithms must transparently reveal decision-making processes and be understandable by clinical experts.

## Data availability statement

The original contributions presented in the study are included in the article/**Supplementary Material**. Further inquiries can be directed to the corresponding author.

## Ethics statement

Ethical approval was not required for the studies on humans in accordance with the local legislation and institutional requirements because only commercially available established cell lines were used.

## Author contributions

RD: Methodology, Software, Validation, Writing – original draft. BY: Conceptualization, Formal Analysis, Writing – review & editing.
