# Supplementary material for: Histopathology image classification: highlighting the gap between manual analysis and AI automation
Source: Front Oncol. 2024 Jan 17;13:1325271. doi: 10.3389/fonc.2023.1325271 (PMC10827850; doi:10.3389/fonc.2023.1325271)
Supplement: Supplementary file 1 [file DataSheet_1.pdf]

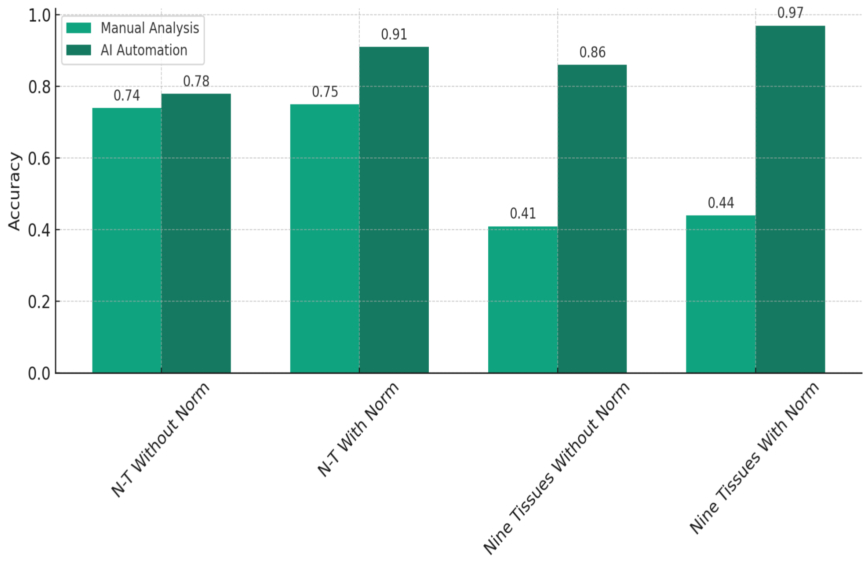

Supplementary Figure 1. Accuracy results (N: Normal, T: Tumor)

The accuracy values from Table 5 are displayed in the bar graph in Supplementary Figure 1. It evaluates the precision of AI automation versus manual analysis in identifying nine tissues and distinguishing between normal and tumor, both with and without normalization. Supplementary Figure 1 makes it evident that for both tissue classifications, AI automation generally performs better in terms of accuracy than manual analysis. Normalization increases accuracy in AI automation more than in manual analysis.

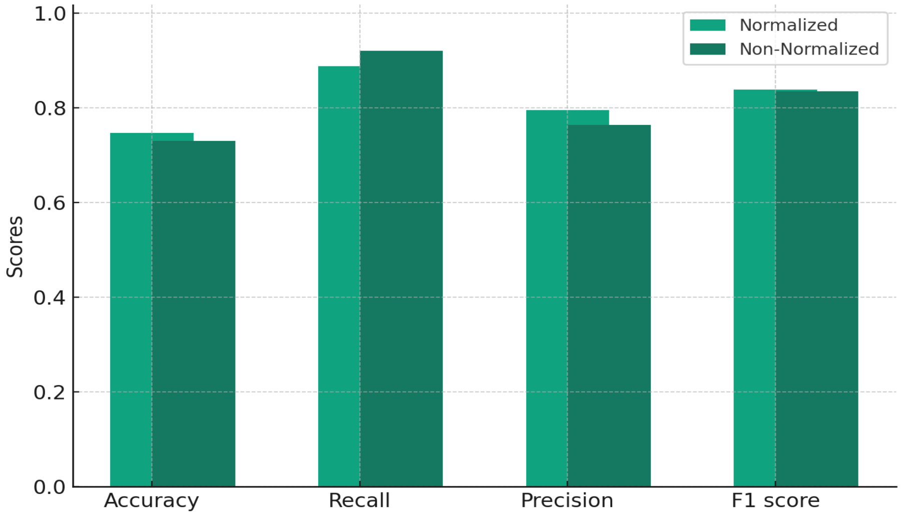

Supplementary Figure 2. Normal and tumor classification evaluation metrics with and without normalization using manual analysis

Table 6 and visual representation Supplementary Figure 2 compares the classification results performed by manual analysis with the effect of normalization.

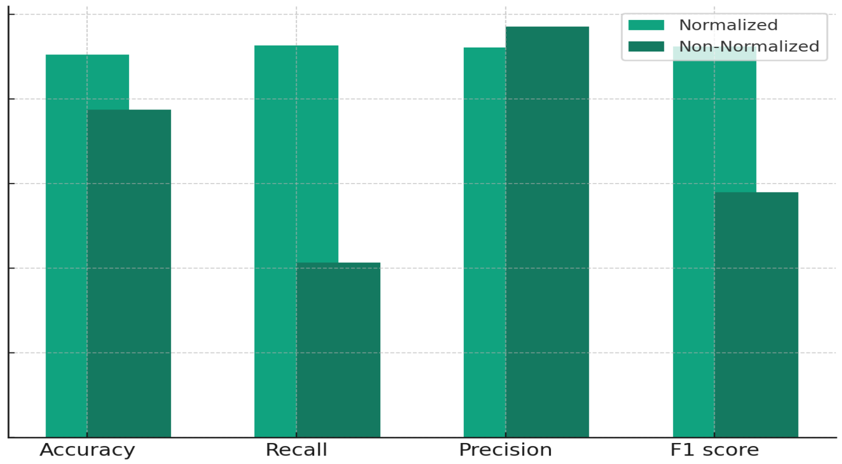

Supplementary Figure 3. The performance metrics of AI automation in classifying normal and tumor tissues with and without normalization

The metrics from Table 7 are displayed in the bar graph in Supplementary Figure 3. It evaluates the effect of with and without normalization in AI automation, distinguishing between normal and tumor. Supplementary Figure 3 shows that normalization in AI automation generally performs better in terms of accuracy, recall, and F1-score than without normalization. Normalization significantly improves these metrics in AI automation more than without normalization.

The confusion matrix was obtained in the study with the "confusionchart" function created using MATLAB R2023a. The confusion matrix shows the relationship between true labels and predicted labels. In Supplementary Figures 4 and 5, precision values are expressed in a row-normalized summary from the confusion matrix, and recall values are expressed in a column-normalized summary. These values allow a more detailed evaluation of the classifier's performance for each class. These metrics are presented more specifically in the main text tables.

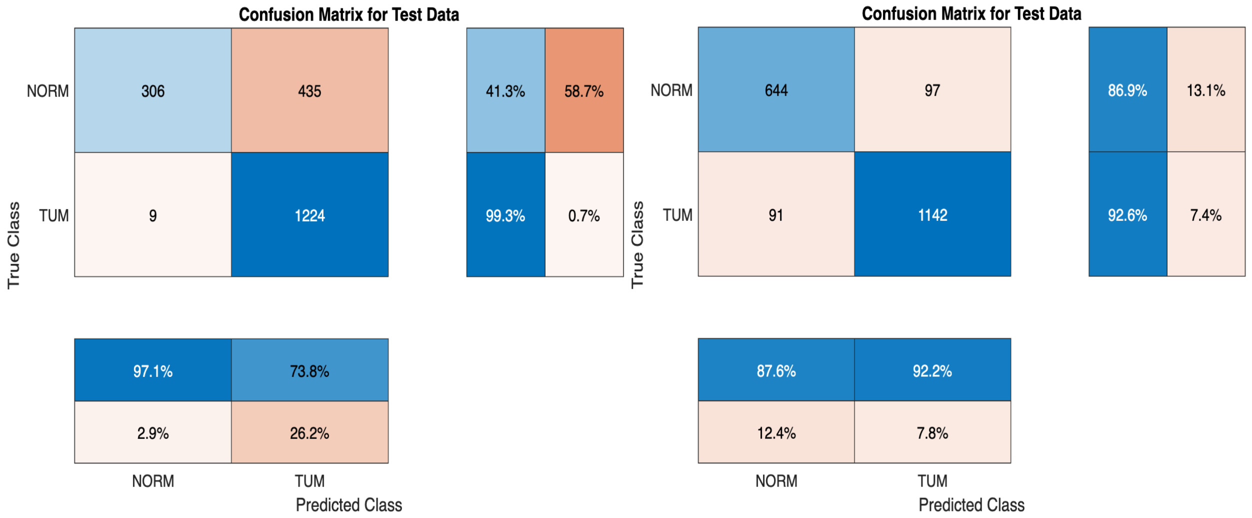

Supplementary Figure 4. Confusion matrix of classifying two different tissues using AI automation a) without and b) with normalization

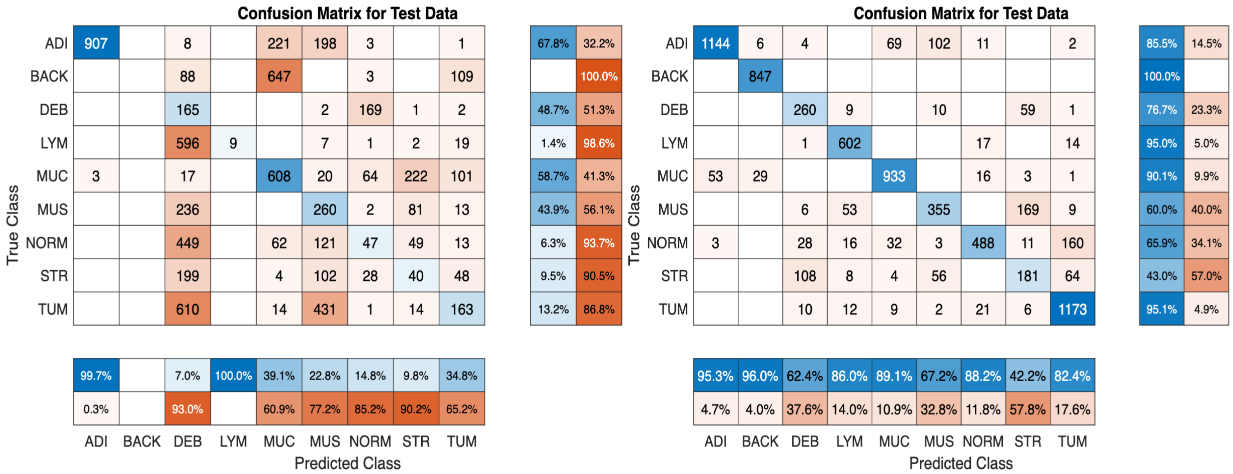

Supplementary Figure 5. a) Confusion matrix of classifying nine different tissues using AI automation without normalization, b) with normalization

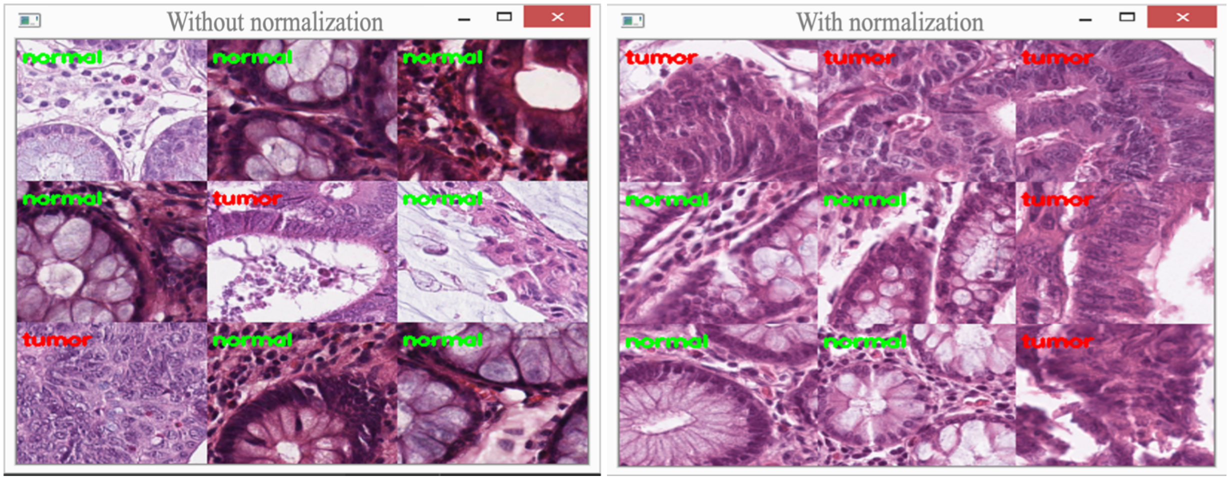

Supplementary Figure 6. Visual results of classifying two different tissues (normal and tumor) a) without normalization and b) with normalization

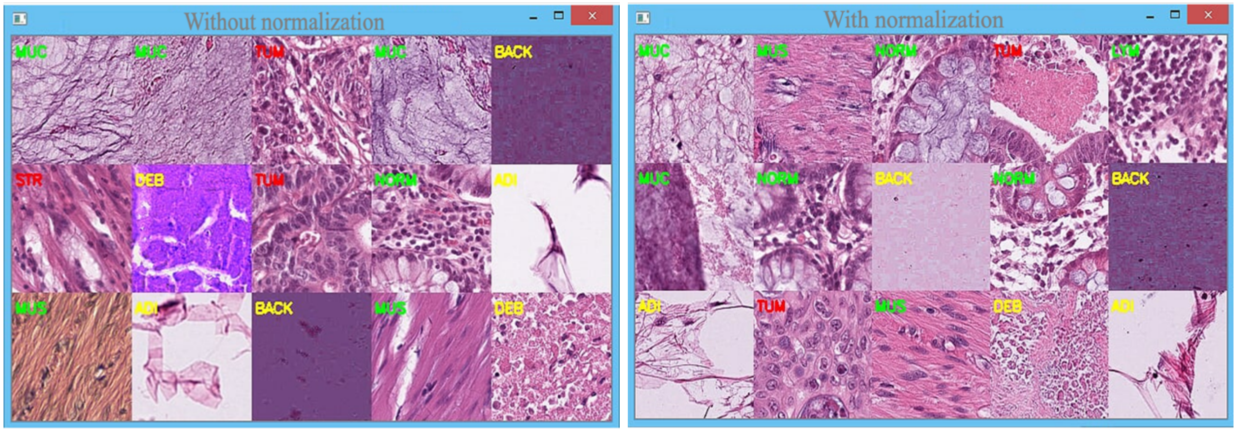

Supplementary Figure 7. Visual results of classifying nine different tissues a) without normalization and b) with normalization
